# Supplementary material for: Cohesin-dependence of neuronal gene expression relates to chromatin loop length
Source: eLife. 2022 Apr 26;11:e76539. doi: 10.7554/eLife.76539 (PMC9106336; doi:10.7554/eLife.76539)
Supplement: Transparent reporting form [file elife-76539-transrepform1.docx]

***eLife’s* transparent reporting form**

We encourage authors to provide detailed information *within their submission* to facilitate the interpretation and replication of experiments. Authors can upload supporting documentation to indicate the use of appropriate reporting guidelines for health-related research (see [EQUATOR Network](http://www.equator-network.org/%20)), life science research (see the [BioSharing Information Resource](https://biosharing.org/)), or the [ARRIVE guidelines](http://www.plosbiology.org/article/info:doi/10.1371/journal.pbio.1000412) for reporting work involving animal research. Where applicable, authors should refer to any relevant reporting standards documents in this form.

If you have any questions, please consult our Journal Policies and/or contact us: [editorial@elifesciences.org](mailto:editorial@elifesciences.org).

**Sample-size estimation**

- You should state whether an appropriate sample size was computed when the study was being designed
- You should state the statistical method of sample size computation and any required assumptions
- If no explicit power analysis was used, you should describe how you decided what sample (replicate) size (number) to use

Please outline where this information can be found within the submission (e.g., sections or figure legends), or explain why this information doesn’t apply to your submission:

No statistical method was used to predetermine the 5C experiment sample sizes. For each experimental condition, 2 replicates of 5C were performed. We selected the number of replicates for 5C based on precedence in the literature and the number of samples we could reasonably perform under financial and logistical constraints to provide accurate estimates of the data's central tendency and variance.

No statistical test was used to determine sample size. Three or more biological replicates per group (as indicated in the figure legends) were used to provide sufficient data to allow statistical inference based on precedence in the literature.

**Replicates**

- You should report how often each experiment was performed
- You should include a definition of biological versus technical replication
- The data obtained should be provided and sufficient information should be provided to indicate the number of independent biological and/or technical replicates
- If you encountered any outliers, you should describe how these were handled
- Criteria for exclusion/inclusion of data should be clearly stated
- High-throughput sequence data should be uploaded before submission, with a private link for reviewers provided (these are available from both GEO and ArrayExpress)

Please outline where this information can be found within the submission (e.g., sections or figure legends), or explain why this information doesn’t apply to your submission:

Biological replicates were generated from independent animals, whereas technical replicates, where used, were repeated experiments from the same biological replicate. Number of replicates is indicated in the figure legends of each experiment.

5C libraries across replicates and conditions within an experiment were prepared, sequenced, and analyzed together. The two 5C experiments (activity induction and cohesin re-expression) were processed independently. All 5C replicates were biological replicates, i.e. prepared from independent cell pellets from independent animals.

**Statistical reporting**

- Statistical analysis methods should be described and justified
- Raw data should be presented in figures whenever informative to do so (typically when N per group is less than 10)
- For each experiment, you should identify the statistical tests used, exact values of N, definitions of center, methods of multiple test correction, and dispersion and precision measures (e.g., mean, median, SD, SEM, confidence intervals; and, for the major substantive results, a measure of effect size (e.g., Pearson's r, Cohen's d)
- Report exact p-values wherever possible alongside the summary statistics and 95% confidence intervals. These should be reported for all key questions and not only when the p-value is less than 0.05.

Please outline where this information can be found within the submission (e.g., sections or figure legends), or explain why this information doesn’t apply to your submission:

Details relating to statistical analysis can be found in figure legends as well as respective sections in methods.

Details regarding the processing of raw 5C reads to loop strength quantifications can be found in the ‘5C Interaction Analysis’ section of the methods.

(For large datasets, or papers with a very large number of statistical tests, you may upload a single table file with tests, Ns, etc., with reference to sections in the manuscript.)

**Group allocation**

- Indicate how samples were allocated into experimental groups (in the case of clinical studies, please specify allocation to treatment method); if randomization was used, please also state if restricted randomization was applied
- Indicate if masking was used during group allocation, data collection and/or data analysis

Please outline where this information can be found within the submission (e.g., sections or figure legends), or explain why this information doesn’t apply to your submission:

No masking was used in experiments as masking was not relevant to this study because we used quantitative assays for all experiments and the computational framework was identical for all samples and replicates.

No masking was used in the 5C experimental process, all replicates were processed together and identically through molecular biology and computational steps.

**Additional data files (“source data”)**

- We encourage you to upload relevant additional data files, such as numerical data that are represented as a graph in a figure, or as a summary table
- Where provided, these should be in the most useful format, and they can be uploaded as “Source data” files linked to a main figure or table
- Include model definition files including the full list of parameters used
- Include code used for data analysis (e.g., R, MatLab)
- Avoid stating that data files are “available upon request”

Please indicate the figures or tables for which source data files have been provided:

Source data have been provided for all Figures
